# National Identity Development Among Minority Youth: Longitudinal Relations with National Fit Perceptions and School Belonging

**DOI:** 10.1007/s10964-024-02036-0

**Published:** 2024-06-19

**Authors:** Nadya Gharaei, Fenella Fleischmann, Karen Phalet

**Affiliations:** 1 German Centre for Integration and Migration Research (DeZIM), Mauerstraße 76, 10117 Berlin, Germany; 2 Center for Social and Cultural Psychology, University of Leuven, Tiensestraat 102—box 3727, 3000 Leuven, Belgium; 3 Department of Sociology, University of Amsterdam, Postbus 15508, 1001 NA Amsterdam, The Netherlands

**Keywords:** Europe, National identification, Perceived fit, Cultural diversity, School belonging, Minority youth

## Abstract

Across Western Europe, immigrant-origin minority youth often struggle to belong socially and to develop national self-identification. Yet, almost no research to-date has asked how these youth perceive the cultural contents of the national identity in their residence country—or rather, to what extent they perceive youth like them to (mis)fit the national identity. The present study addressed this research gap by centering schools as developmental contexts of evolving belonging and national self-identification and newly inquiring into minority youth’s perceptions of national (mis)fit as critical levers of their national identity development. Drawing on data from two annual waves of the Leuven-Children of Immigrants Longitudinal Study (Leuven-CILS), a sample of 942 Moroccan- and Turkish-origin youth (*M*_age-T1_ = 14.98, SD = 1.22; 52% female) in 62 Belgian schools was used. Cross-lagged analysis combined repeated measures of school belonging and national self-identification with vignette measures of the perceived national fit of (imagined) culturally different peers. While school belonging and national self-identification were unrelated over time, earlier perceived national fit uniquely enabled more national self-identification one year later, over and above evolving school belonging. These findings suggest that experiencing belonging in school does not suffice for minority youth to develop national self-identification. Schools may, however, promote national identity development through redefining national identities to include cultural diversity—thereby signaling to minority youth that they can fit the national identity.

## Introduction

Across Western Europe, immigrant-origin minority youth^1^ often struggle to belong socially and to develop national self-identification (Fleischmann & Phalet, 2018). National self-identification captures how strongly individuals feel an internalized sense of belonging to their national community (Umaña-Taylor et al., 2020) and as such is known to promote psychological well-being (Khan et al., 2020) and civic engagement (Fleischmann et al., 2013). Yet, past research on minority youth’s identity development has focused mostly on their ethnic or racial identities (Umaña-Taylor et al., 2014). More recently, research work has started to inquire into their national or dual (i.e., ethnic and national) identity development (Fleischmann et al., 2019; Leszczensky et al., 2019; Spiegler et al., 2018, 2019; Umaña-Taylor et al., 2020); however, this work has focused narrowly on (changes in) strength of self-identification without asking how these youth view the national identity contents of the country in which they are growing up. This lack of research into minority youth’s perceptions of national identity contents mirrors social reality, where minority views on a common national identity are generally less visible and where their claims on this identity are contested (Moffitt & Juang, 2019; Syed et al., 2018). The present study investigated how minority youth perceive the contents of the national identity and how their perceptions of cultural difference and national (mis)fit in the school context inform their national identity development over and above evolving school belonging.

National fit perceptions refer to a subjective (mis)match with the perceived contents of the national identity and in this study capture the extent to which minority youth perceive the contents of the national identity to be open to cultural diversity—or rather, to cultural difference from the non-immigrant majority group. As national identities are culturally informed constructs (Ashmore et al., 2004), their contents are defined “in the form of intersubjective perceptions of culture—beliefs and values that members of a culture perceive to be widespread in their culture” (Chiu et al., 2010, p. 482). Importantly, these contents are not viewed as essentially fixed and consensually endorsed but rather as emergent and continuously (re)defined in social interactions with others (Chiu et al., 2010). A previous cross-sectional study validated the intersubjective nature of national identity contents: Its empirical findings showed that minority youth varied in their national fit perceptions of culturally different peers and that the minority youth attributed greater national fit to these peers when they perceived other nationals to be more accepting of them (Gharaei et al., 2018).

In keeping with national fit perceptions as socially grounded, a key developmental context where minority adolescents negotiate cultural difference and national fit in social interactions with others is the school environment (Umaña-Taylor et al., 2020). As educational institutions, all schools transmit cultural and sociopolitical knowledge, beliefs, and practices as part of their mission to prepare students to become competent and active citizens (Council of Europe, 2020). At the same time, schools differ considerably in their approaches to cultural diversity, varying from restrictive assimilationism to more inclusive policies valuing cultural difference (Celeste et al., 2019); and, they differ in the ethnic composition of their student body ranging from low to very high presence of immigrant-origin minority students (Gharaei et al., 2018). Consequently, schools provide minority adolescents with variable opportunities for negotiating cultural difference and national fit in social interactions with peers and teachers (Titzmann & Jugert, 2019).

This study focused specifically on Moroccan- and Turkish-origin minority youth in Belgian secondary schools. Moroccan- and Turkish-origin youth represent two of the most well-represented and predominantly Muslim minority groups in the Belgian and European context (Heath & Brinbaum, 2014). In fact, it has been estimated that about 2% of the Belgian population is of Turkish origin (Schoonvaere, 2013) and about 3.8% of Moroccan origin (Schoonvaere, 2014). Their presence in Belgium, and Europe more generally, originated in the labor migration of the 1960s and subsequent family reunifications (Gsir et al., 2015). Although most Moroccan- and Turkish-Belgian youth today are 2nd or 3rd generation immigrants and hold formal Belgian national citizenship (Gsir et al., 2015), they continue to face persistent structural disadvantages and pervasive prejudice (Heath & Brinbaum, 2014). For instance, like other European countries, Belgium is characterized by relatively high levels of neighborhood and school segregation (Andersson et al., 2018; Baysu & de Valk, 2012), meaning that Moroccan- and Turkish-Belgian youth often live in low-income neighborhoods or attend schools where non-EU immigrant-origin and predominantly Muslim minority persons or students are overrepresented. In general, Moroccan- and Turkish-origin youth face a rise in Islamophobia in Belgium and Europe today (Dikici, 2020), with their affiliation with Islam considered a significant ethno-religious marker of cultural difference from the majority group that serves to exclude them from the national group (Alba & Foner, 2015). Along those lines, previous research has shown that Muslim minority youth in Europe report even lower levels of national self-identification than other non-Muslim minority youth do (Leszczensky et al., 2020).

### A Minority Perspective on National Identity

National membership represents a psychologically significant common identity not only for non-immigrant majority but also for immigrant-origin minority adolescents (Wang et al., 2015). Immigrant-origin minority youth typically care about national membership in their country of residence. For instance, qualitative research that conducted in-depth interviews with Moroccan-Dutch young adults revealed that for them their bond with the Netherlands felt more natural than that with Morocco (Omlo, 2011). Not only did they describe the Netherlands as their home country, they also wished to contribute to Dutch society and to build their future in the Netherlands. At the same time, they shared experiences of ethnic discrimination in their daily social interactions and were aware that Dutch society did not view them as “being Dutch” regardless of their efforts to integrate; and these experiences of ethnic exclusion made it harder for them to claim their being or feeling Dutch. Similar minority narratives of contested national belonging emerged in qualitative interviews in, for instance, Germany (Moffitt et al., 2018), Austria (Vietze et al., 2018) and Denmark (Simonsen, 2018). Together, these case studies convey minority persons’ struggle to self-identify and be recognized as fellow nationals while being (seen as) culturally different from majority nationals.

In Western European countries today, a “true” national tends to be defined by the national majority group as someone who shares a common ancestry, who speaks the national language(s), and who endorses majority (Christian) faith and cultural traditions (Pehrson et al., 2009; Reijerse et al., 2013). In line with a prevailing cultural representation of national identity in Europe, a “true” national is commonly defined as someone who values and wishes to preserve a national culture in line with the cultural norms, customs, and traditions of the dominant majority group (Reijerse et al. 2013). Through defining national identity contents in exclusively majority cultural terms, majorities delineate the cultural boundaries of the nation in a way that excludes minority nationals who are (seen as) culturally different from them. Thus, even when they are formally recognized as national citizens, minority persons may find themselves at the margins of national membership due to their cultural difference from the majority group (Simonsen, 2018).

Importantly, quantitative studies on the contents of the national identity from a majority group perspective (e.g., Duriez et al., 2013; Pehrson & Green, 2010) showcase multiple socially shared definitions of the same national identity. This variation in national identity definitions resonates with an intersubjective approach to national identities, which centers social embeddedness as well as content and meaning as constitutive elements of collective identification (Chiu et al., 2010). To be psychologically viable, national identity definitions need not be consensual, yet meaningful identity contents need to be socially (re)negotiated and validated (Gharaei et al., 2018). Accordingly, identity definitions are reproduced or challenged in public discourse (e.g., by educational institutions) and in everyday interactions (e.g., with teachers or peers), giving rise to multiple alternate identity definitions.

Yet, minority voices have been largely absent from national identity discourse in Western Europe until recently. One reason why minority voices are too often still missing in public debates, is the continued under-representation of immigrant-origin minority persons in national institutions and on the political stage (Bloemraad & Schönwälder, 2013; Ecevit & Kinsey, 2022). Consequently, minority groups often lack representatives who publicly embody and thus might collectively redefine the national identity in ways that include cultural diversity. Against the backdrop of enduring power asymmetries and ensuing reality constraints on alternate identity definitions at the national level, a central question is how minority youth negotiate cultural difference and national fit from the bottom up in their day-to-day engagement in the school setting.

### Examining National Fit Perceptions

Cultural socialization in European schools today tends to refer exclusively to majority cultural knowledge, beliefs, norms, and practices (Celeste et al., 2019), thus explicitly or implicitly defining the national identity in ways that exclude cultural difference. Yet, research to-date has still to ask what role cultural difference and varying perceptions of national fit in the school context play for minority youth’s national identity development.

National fit perceptions can inform national self-identification via a cognitive process of *self-anchoring* by which individuals mentally connect the self to the nation (Van Veelen et al., 2016). Social identification has traditionally been explained as a product of self-stereotyping, whereby individuals align the self with the group by highlighting personal characteristics that fit with the group prototype (i.e., “I am like the group”). In contrast, self-anchoring aligns the self with the group by selectively redefining the group to reflect one’s personal characteristics (i.e., “The group is like me”). Rather than being mutually exclusive, both processes are found to co-occur, thus complementing each other in enabling social identification (Van Veelen et al., 2016). Accordingly, minority youth achieve national self-identification by aligning the self with prototypical characteristics in prevailing national identity definitions (i.e., self-stereotyping) and by redefining the cultural contents of the national identity to include the self (i.e., self-anchoring).

Since prototypical characteristics in prevailing European national identity definitions are to-date exclusively *majority cultural* characteristics (Reijerse et al., 2013), achieving national self-identification via self-stereotyping for minority youth in Europe today means to pursue the acculturation strategy of assimilation (Berry et al., 2006)—or rather, to assimilate to the majority culture and leave their distinct heritage culture(s) behind. Accordingly, previous research has shown that those who assimilate to the majority culture, also self-identify more strongly as a national (Verkuyten & Martinovic, 2012). In contrast, achieving national self-identification via self-anchoring shifts the explanatory focus from individual acculturation strategies in mainstream acculturation research to how European national identities can be redefined to include cultural diversity (Kunst et al., 2021). Specifically, perceiving culturally different peers to fit the national identity indicates that socially shared definitions of the national identity include cultural diversity, thereby *allowing* minority youth to develop national self-identification via self-anchoring. Only when the intersubjective contents of the national identity are open to cultural diversity, minority youth can self-anchor aspects of their distinct heritage culture(s) in the national identity to achieve national self-identification. Accordingly, Moroccan- and Turkish-Belgian youth in an earlier cross-sectional study indeed self-identified more strongly as Belgian nationals, the more they perceived culturally different peers to fit the national identity (Gharaei et al., 2018).

### Tracing School Belonging and National Self-Identification

The role of school belonging for students’ academic and psychological adjustment has been well-established (Allen et al., 2018), also for minority students (e.g., Celeste et al., 2019; Heikamp et al., 2020). However, minority youth’s school belonging and national self-identification have largely been studied in separate research streams, so that little is known about how they develop in conjunction.

School belonging has been defined as “the extent to which students feel personally accepted, respected, included, and supported by others in the school social environment” (Goodenow & Grady, 1993, p. 80). Indeed, known key predictors of students’ feelings of belonging in school are positive interpersonal relations with teachers and peers (Allen et al., 2018). As these interpersonal relations are often intercultural encounters from the perspective of minority students, school belonging can be viewed as an indicator of social belonging through positive intercultural relations (Baysu et al., 2021). Against this background, the social belonging of minority adolescents is grounded in the school environment.

Schools are embedded in the wider social and cultural context; and, minority youth who experience a sense of belonging in school may by extension form an attachment to the nation and vice versa. Ideally, membership in the nation as imagined community (Anderson, 1983) becomes tangible or real through adolescents’ day-to-day engagement with other nationals in local social settings such as the school. Moreover, schools are national institutions^2^ with a formal mission to educate students into competent and active citizens (Council of Europe, 2020). Not only is teaching of national languages, literatures, and history a significant part of the explicit school curriculum, schools also informally communicate sociopolitical knowledge, beliefs, and values that are prevalent in the wider society; and school activities routinely require students’ active participation in national traditions (e.g., sport competitions, celebration of national holidays).

## The Current Study

Little is known about how minority youth perceive the national identity contents of the country in which they are growing up. In line with an intersubjective approach to identity contents as socially grounded and culturally informed, minority youth’s perceptions of cultural difference and national fit were examined in their day-to-day-school environment and longitudinally related to their national identity development. To this end, national fit perceptions were operationalized in this study as a function of cultural difference from the majority group using validated vignette measures of (imagined) varying culturally different peers. In accordance with national self-identification via self-anchoring, minority youth who perceived imagined culturally different peers to fit the national identity better at time 1 were expected to increase in their national self-identification over one year. Since schools are embedded in the nation as national institutions, this study also accounted for the youth’s evolving school belonging and newly explored whether school belonging and national self-identification may be mutually reinforcing over time. In addition, minority youth’s own acculturation attitudes were controlled for to account for national self-identification via self-stereotyping in the form of adopting the majority culture.

## Methods

### Data

Large-scale longitudinal survey data from the Leuven-CILS project, collected in ethnically diverse secondary schools in Flanders, Belgium, was used. The Leuven-CILS project is modeled on and affiliated with the Children of Immigrants Longitudinal Survey in Europe (CILS4EU; Kalter et al., 2016). Detailed information on the Belgian samples, constructs, and measures can be found in the Leuven-CILS technical report (Phalet et al., 2018; available upon request). In line with the CILS4EU stratified random sampling design, school-level administrative information on foreign languages spoken at home was used to randomly select *n* = 70 Belgian secondary schools with varying shares of immigrant-origin minority students (<10%, 10–30%, 30–60%, >60%). Within these schools, students from randomly selected classes in grades 1–3 filled out a paper-and-pencil questionnaire three times, in three consecutive years; the first wave of data collection took place in the academic year 2012/2013 (time 1), followed by further data collections in 2013/2014 (time 2) and 2014/2015 (time 3). The questionnaires were administered in Dutch as the school language and national language in Flanders, Belgium. Prior to the administration of the questionnaires during class hours, informed consent from school principals, teachers, participants, and their parents was obtained. Participation was voluntary and students could drop out at any time. Moreover, anonymity of the students was guaranteed by assigning each student a unique identification number and stripping the files of the collected survey data of any information that would allow identifying individual students (or their classroom or school).

### Participants

The present study draws on the Leuven-CILS survey data (cf. supra) from Moroccan- and Turkish-Belgian subsamples specifically. To maximize sample size and thus the statistical power of analyses, only Leuven-CILS data from wave 1 and wave 2 were used. First, *N* = 1508 Moroccan- or Turkish-origin students in the total time 1 sample of *N* = 5336 students in *n* = 70 secondary schools were identified based on self-reported parentage (i.e., those who reported to have at least one parent or two grandparents born in Morocco or Turkey). Then the study sample was narrowed down to those who filled out the survey at both time 1 and time 2, leaving a final study sample of *N* = 942 youth of Moroccan (*N* = 514) or Turkish (*N* = 428) origin in *n* = 278 classrooms across 62 secondary schools in Flanders, Belgium. Demographics for the study sample and the Moroccan- and Turkish-origin subsamples are shown in Table 1. Most of the youth participants in the study sample were 2nd generation (75%) and self-categorized as Muslim (85%); their ages at time 1 ranged from 12 to 19 (*M*_age-T1_ = 14.98, SD = 1.22), and 52% were female. As Table 1 shows, similar distributions were found across the Moroccan- and Turkish-origin subsamples. Moreover, almost half of the youth in the sample (48%) were situated in schools with more than 60% immigrant-origin minority students in school, while 35% were in schools with 30–60% and only 16% in schools with less than 30% minority students. However, a larger share of the Moroccan- than Turkish-origin participants (55% vs. 40%) were situated in schools with more than 60% minority students in school.

**Table 1:** Demographics for full study sample and Moroccan- and Turkish-origin subsamples

	Study sample (*N* = 942)	Moroccan-origin subsample (*N* = 514)	Turkish-origin subsample (*N* = 428)
Age _T1_	14.98 (1.22)	14.97 (1.24)	14.99 (1.20)
Girls	52%	53%	52%
2nd generation	75%	72%	79%
Self-categorization as Muslim	85%	86%	84%
Ethnic school composition _T1_			
>60% minority in school	48%	55%	40%
30–60% minority in school	35%	34%	36%
<30% minority in school	16%	11%	23%

Means (standard deviations in parentheses). 2nd generation = youth born in Belgium with at least one parent born in Morocco or Turkey. Ethnic school composition = the percentage of immigrant-origin minority students in school based on school-level administrative information on foreign languages spoken at home

### Measures

Repeated measures for national self-identification and school belonging were based on identical item wordings and response categories.

#### National self-identification

National self-identification was assessed with the question “How strongly do you feel Belgian?” rated on a scale ranging from 1 (I don’t feel Belgian) to 5 (very strongly). This measure of national self-identification has been used successfully in previous research with immigrant-origin Muslim and other minority youth in Belgium, England, Germany, the Netherlands and Sweden (Fleischmann & Phalet, 2018) and is in line with the established validity of single-item measures of social identification more generally (Postmes et al., 2013). Nevertheless, an additional analysis was conducted (cf. infra) to correct for measurement error in the single-item measure of national self-identification.

#### School belonging

A sense of belonging at school was assessed with four items (adapted from Wang et al., 2011) that have been validated in previous research with Moroccan- and Turkish-origin youth in Belgium (Baysu et al., 2021; Heikamp et al., 2020). The youth participants responded on a scale from 1 (strongly disagree) to 5 (strongly agree) to the following statements: (1) “I am proud to be a student in this school”, (2) “I would prefer to go to another school” (reverse coded), (3) “I feel happy in this school” and (4) “I feel at home in this school”. The four items for school belonging formed reliable scales at time 1 and time 2 (*α* = 0.83 and *α* = 0.85, respectively).

Moreover, the construct validity of the school belonging measure was corroborated by expected within-time associations with measures of teacher support (e.g., “Teachers understand you”; time 1 *r* = 0.386, *p* < 0.001, time 2 *r* = 0.390, *p* < 0.001) and peer rejection (e.g., “How often do you experience other students excluding you?”; time 1 *r* = −0.216, *p* < 0.001, time 2 *r* = −0.132, *p* < 0.001) as external correlates. These correlations show that the youth’s sense of belonging in school has a relational dimension that is indicative of social belonging.

#### Perceived national fit of imagined culturally different peers

**Fig. 1:**
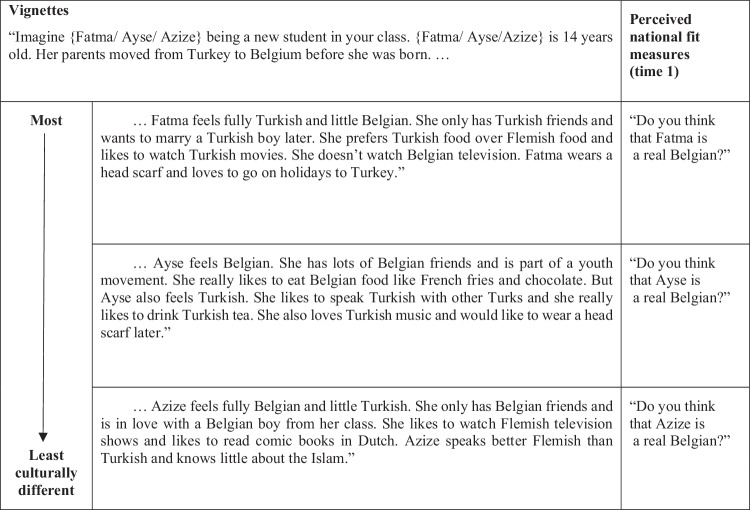
Within-subject vignette measures of the perceived national fit of imagined culturally different peers. All three measures were rated on a five-point scale (1 = absolutely not, 5 = very much); each represents a single-item measure of the perceived national fit of *most*, *less*, and *least* culturally different peers, respectively. The term “Flemish” in the vignettes refers to the Dutch language and customs in the Dutch-speaking part of Belgium (known as Flanders or the Flemish Region) where data collection for this study was conducted

Measures of the perceived national fit of imagined culturally different peers were only available at time 1 and consist of three single-indicator vignette measure that captured the perceived national fit of *most*, *less* and *least* culturally different peers, respectively (see Fig. 1). The three vignette measures have been validated in a previous cross-sectional study (Gharaei et al., 2018) and assess perceived national fit as a function of cultural difference from the majority group—namely, by varying cultural difference across three vignettes describing imaginary peers. The perceived national fit of the imagined culturally different peers was measured with the question “Do you think that … is a real Belgian?” Participants answered the question on a scale from 1 (absolutely not) to 5 (very much); responses indicate to what extent they perceived the imagined peers to fit the national identity.

As the wordings of the three vignettes in Fig. 1 show, the described peer named Fatma is *most* culturally different from majority Belgians as she is maintaining the customs of her heritage culture while not adopting the customs of the majority culture. *Less* culturally different than Fatma is the described peer named Ayse who is oriented toward both the majority and her heritage culture. Lastly, the described peer named Azize is the *least* culturally different of the three as she is described as having adopted customs of the majority culture while not maintaining the customs of her heritage culture. Gender was kept constant across the vignettes; similarly, common Muslim names were used, and specific cultural contents were slightly different between the vignettes to enhance face validity in a within-subject design. Note that further information on how the vignette measures were developed can be found in the online supplementary materials. By means of the developed vignettes, the perceived national fit of imagined peers was measured rather than the youth’s self-perception of their own national fit; thus, the vignette measures of this study avoid self-representation biases (Hughes & Huby, 2002) and resonate with an intersubjective approach (Chiu et al., 2010).

#### Control variables

To estimate net effects of the main predictor variables, *age*, *ethnic origin* (1 = Turkish, 0 = Moroccan), *gender* (1 = girls, 0 = boys) and time 1 *ethnic school composition* were included as statistical controls in the models. Ethnic school composition was operationalized using three dummy variables. The use of dummy variables enabled a comparison of those with more than 60% immigrant-origin minority students in school (reference category) to those who had less than 30% and 30–60% minority students in school, respectively.

In addition, associations of participants’ own acculturation attitudes at time 1 with their national self-identification and school belonging at time 1 and time 2 were controlled for. This was done (a) to disentangle the Moroccan- and Turkish-origin youth’s responses on the perceived national fit measures (cf. supra) from their own acculturation attitudes and (b) to account for national self-identification via self-stereotyping in the form of adopting the majority culture. Previous research has shown that those who report favoring majority culture adoption over heritage culture maintenance, and thus assimilate to the majority culture, self-identify more strongly as a national (Verkuyten & Martinovic, 2012). Thus, the youth’s own acculturation attitudes were measured with commonly used general statements about majority culture adoption and heritage culture maintenance (Arends-Tóth & van de Vijver, 2006). On a scale from 1 (strongly disagree) to 5 (strongly agree), participants rated the statements “Migrants should adopt the Belgian customs in this country” (*own attitude toward majority culture adoption; M* = 2.97, SD = 1.24) and “Migrants should do everything possible to preserve the customs of their country of origin” (*own attitude toward heritage culture maintenance; M* = 3.46, SD = 1.12).

### Analyses

**Fig. 2:**
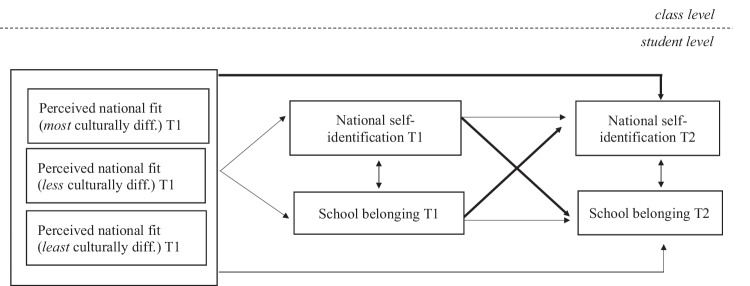
Specified associations between the main study variables. Lines drawn from the box on the left each represent three associations with time 1 perceived national fit of imagined *most*, *less*, and *least* culturally different peers, respectively. Associations pertaining to the main associations of interest are shown in bold. The three time 1 measures of the perceived national fit of *most*, *less* and *least* culturally different peers were allowed to co-vary. All associations were controlled for age, gender, ethnic origin, own acculturation attitudes and ethnic school composition (all measured at time 1; controls not shown). diff. = different

For the main analyses, a longitudinal cross-lagged model in Mplus 8 (Muthén & Muthén, 1998–2017) with repeated measures of national self-identification and school belonging was tested. In this model, the nested structure of the data was accounted for by specifying time 1 school classes as clusters (*n* = 278). Figure 2 shows the specified associations between the main study variables in the model; all associations were controlled for age, gender, ethnic origin, and own acculturation attitudes at the student level and ethnic school composition at the class level. In the model, the three within-subject measures of perceived national fit were allowed to covary. To deal with missing data, full information maximum likelihood (FIML) estimation (Enders & Bandalos, 2001) was used, an approach suitable also for nested data (Larson, 2011).

To test the hypothesis about the over-time relation between perceptions of national fit and national self-identification, national self-identification at time 2 was regressed on the three measures of perceived national fit (i.e., the perceived fit of the imagined *most*, *less*, and *least* culturally different peer, respectively). Note that this was done while controlling for change in national self-identification (stability path 1) and time 1 school belonging.

To explore whether school belonging and national self-identification may mutually reinforce each other over time, the relations between time 1 and time 2 school belonging (stability path 2) were specified as well, and the national self-identification and school belonging measures were allowed to co-vary within time. Controlling for these stability paths and within-time relations, the relations between time 1 school belonging and time 2 national self-identification (cross path 1), and time 1 national self-identification and time 2 school belonging (cross path 2) were estimated.

Given that little is known about whether and how minority youth’s national self-identification, perceptions of national fit and school belonging are related, associations that go beyond the theoretical expectations of this study were explored as well. For this reason, a fully symmetrical model was specified that also included associations of the three time 1 perceived national fit measures with (a) time 1 national self-identification and time 1 school belonging and (b) time 2 school belonging.

## Results

### Attrition Analysis

Moroccan- and Turkish-origin students who took part in the survey at both time 1 and time 2 (i.e., the study sample; *N* = 942) were compared to those who participated only at time 1 (drop-out sample; *N* = 566). The rather high drop-out rate is predominantly because over one year students moved to different classes (based on their track choices) or to new schools that were beyond the reach of data collection during the second wave. In the attrition analysis, two-sample t-tests were used to compare the time 1 means of continuous variables (age, national self-identification, national fit perceptions, school belonging and own acculturation attitudes) across the study and drop-out sample. For the dichotomous variables of gender (1 = girls, 0 = boys) and ethnic origin (1 = Turkish origin, 0 = Moroccan origin), percentages were compared across the two samples of interest.

Across the study and drop-out sample, the shares of Turkish-origin youth were similar (45% and 44% Turkish-origin youth, respectively). However, compared to the study sample, the drop-out sample consisted of a lower share of girls (37% compared to 52% in the study sample) and somewhat older participants, *t*(1436) = −3.774, *p* < 0.001. Moreover, participants in the drop-out sample reported somewhat lower school belonging than those in the study sample, *t*(1455) = 6.244, *p* < 0.001. Only marginally significant differences between the study and drop-out sample were found for national fit perceptions: Participants in the drop-out sample perceived slightly greater national fit of the imagined *least* culturally different, *t*(1450) = −1.756, *p* = 0.078, as well as the imagined *most* culturally different peer, *t*(1455) = −1.907, *p* = 0.057, than those in the study sample. No significant differences between the study and the drop-out sample were found in national self-identification, perceived national fit of the imagined *less* (i.e., in-between most and least) culturally different peer and own acculturation attitudes. Thus, while this study was able to reach a large sample of Moroccan- and Turkish-origin students in Belgian secondary schools, it is important to keep in mind that the dropout from this sample was not entirely random. In particular, the socio-demographic composition of the sample changed over time, meaning that the results of this study should not be generalized to the student population targeted in wave 1. What is, however, reassuring, is that no selectivity in the dependent variable, namely the national self-identification of the Moroccan- and Turkish-Belgian youth, was found.

### Descriptive Results

Mean scores and standard deviations for the main study variables are presented in Table 2. In addition, bivariate correlations between these variables are reported in Table S1 in the online Supplementary Materials. Overall and as expected, the Moroccan- and Turkish-origin youth participants self-identified as Belgian nationals only moderately. More specifically, the mean of time 1 national self-identification did not significantly differ from the midpoint of its five-point scale (with the midpoint “3” meaning “not so strongly”), while the mean of time 2 national self-identification was only slightly above this midpoint, *t*(868) = 1.891, *p* = 0.059. On average the youth participants’ self-identification with the national group did not significantly change over time.

**Table 2:** Means and standard deviations of the main study variables (*N* = 942)

	Time 1		Time 2	
	*M*	SD	*M*	SD
National self-identification	2.98^a^	1.33	3.08^a^	1.26
School belonging	3.58	0.92	3.46	0.98
Perceived national fit (*most* culturally different)	1.95	1.27	–	–
Perceived national fit (*less* culturally different)	2.55	1.25	–	–
Perceived national fit (*least* culturally different)	3.35	1.37	–	–

All measures were rated on a five-point scale (range 1–5). Perceived national fit measures were only available at time 1

^a^Mean scores do not significantly differ; *t*(804) = −1.375, *p* = 0.170

The Moroccan- and Turkish-origin youth also reported a moderate sense of school belonging. Overall, they did not disagree with statements reflecting school belonging (e.g., “I feel at home in this school”), neither did they strongly agree; at both time points, mean scores of school belonging fell between “3” (neither agree nor disagree) and “4” (agree) on its five-point scale. On average the youth’s school belonging decreased somewhat over time, *t*(900) = 3.767, *p* < 0.001.

Turning to the perceived national fit measures, on average the Moroccan- and Turkish-origin youth perceived the imagined *most* culturally different peer least as a “real” Belgian, indicating low national fit. The mean for the imagined *less* culturally different peer was still significantly below the midpoint of its five-point scale, *t*(899) = −10.733, *p* < 0.001, again indicating relatively low national fit in the eyes of the youth participants. Only the *least* culturally different peer in the vignettes was perceived as a “real” Belgian, with a mean score above the midpoint of the scale, *t*(933) = 7.739, *p* < 0.001. However, the mean score indicated only moderate national fit, suggesting that even assimilation does not make minority youth entirely or unambiguously Belgian. These descriptive results replicate previous findings (Gharaei et al., 2018) and show that the Moroccan- and Turkish-Belgian youth perceived the more culturally different peers to fit the national identity less well than a least culturally different peer. This pattern is in line with prevailing definitions of the national identity in majority cultural terms (Reijerse et al., 2013).

### Perceived National Fit, School Belonging and National Self-Identification

To test the hypothesis about the role of national fit perceptions in the development of Moroccan- and Turkish-origin youth’s national self-identification over and above evolving school belonging, a longitudinal model with repeated measures of national self-identification and school belonging was estimated (see Fig. 2). Overall, model fit indices indicated that this model fits the data reasonably well (CFI = 0.948, TLI = 0.828, RMSEA = 0.041). The model results, while controlling for students nested in school classes, are presented in Table 3; unstandardized regression coefficients with standard errors in parentheses are reported.

**Table 3:** Results of the longitudinal model explaining school belonging and national self-identification at time 1 and time 2 (*N* = 942)

	Time 1		Time 2	
	School belonging	National self- identification	School belonging	National self-identification
	*B* (S.E.)	*B* (S.E.)	*B* (S.E.)	*B* (S.E.)
Individual level				
National self-identification T1	–	–	0.037 (0.030)	0.261 (0.046)***
School belonging T1	–	–	0.425 (0.044)***	0.041 (0.059)
Perceived national fit (*most* culturally different) T1	−0.014 (0.029)	0.139 (0.042)**	0.024 (0.031)	0.092 (0.041)*
Perceived national fit (*less* culturally different) T1	−0.026 (0.027)	0.041 (0.040)	0.003 (0.031)	−0.025 (0.038)
Perceived national fit (*least* culturally different) T1	−0.028 (0.024)	0.007 (0.036)	−0.063 (0.025)*	−0.035 (0.031)
Controls				
Age	−0.084 (0.027)**	−0.036 (0.037)	−0.003 (0.028)	−0.018 (0.036)
Girls	0.178 (0.069)*	0.286 (0.092)**	0.003 (0.060)	0.125 (0.086)
Turkish origin	0.099 (0.070)	0.007 (0.100)	0.086 (0.063)	−0.116 (0.089)
Own attitude toward majority culture adoption T1	0.057 (0.025)*	0.215 (0.037)***	0.059 (0.027)*	0.147 (0.037)***
Own attitude toward heritage culture maintenance T1	0.018 (0.030)	−0.079 (0.043)^†^	0.021 (0.029)	0.014 (0.041)
Class level				
Controls				
>60% minority in school T1 (ref. cat.)	–	–	–	–
30–60% minority in school T1	0.000 (0.086)	0.216 (0.105)*	−0.050 (0.079)	0.126 (0.104)
<30% minority in school T1	0.202 (0.095)*	0.395 (0.126)**	−0.006 (0.105)	0.331 (0.130)*
Model Fit Indices: CFI/TLI/RMSEA	0.948/0.828/0.041			

Unstandardized regression coefficients with standard errors in parentheses are reported. The three vignette measures used to assess perceived national fit (range 1–5) are illustrated in Fig. 1

ref. cat. = reference category

^†^*p* < 0.10; *p < 0.05; **p < 0.01; ***p < 0.001

Model results showed that the Moroccan- and Turkish-origin youth who at time 1 perceived the imagined *most* culturally different peer to fit the national identity more, also self-identified more strongly as a Belgian national both within time, *B* = 0.139, S.E. = 0.042, *p* = 0.001, and over time (i.e., one year later), *B* = 0.092, S.E. = 0.041, *p* = 0.023. No significant within- or over-time relations with national self-identification were found for the perceived national fit of the imagined *less* and *least* culturally different peers. Note that the tested over-time associations between time 1 national fit perceptions and time 2 national self-identification were controlled for time 1 national self-identification.

In addition, whether school belonging and national self-identification may mutually reinforce each other over time was explored. Model results did not reveal significant cross-lagged paths between school belonging and national self-identification: School belonging at time 1 was unrelated to national self-identification at time 2 (while controlling for national self-identification at time 1). Similarly, national self-identification at time 1 was unrelated to school belonging at time 2 (while controlling for school belonging at time 1).

Going beyond main associations of interest, model results also showed that the Moroccan- and Turkish-origin youth who perceived greater national fit of the imagined *least* culturally different peer, reported a decrease in school belonging one year later, *B* = −0.063, S.E. = 0.025, *p* = 0.047. No significant within-time relation between the perceived national fit of the imagined *least* culturally different peer and school belonging was found.

The results regarding the control variables showed that at time 1 girls identified more strongly as Belgian nationals than boys; however, this gender difference in national self-identification disappeared at time 2. Moreover, the youth participants who viewed adopting the majority culture more favorably, also identified more strongly as Belgian nationals both at time 1 and time 2. In contrast, a more favorable attitude toward heritage culture maintenance was only marginally and negatively related to national self-identification at time 1. No significant difference in national self-identification was found between Moroccan- and Turkish-origin participants. Finally, compared to the participants in schools with more than 60% minority students, (a) those in schools with less than 30% minority students reported higher national self-identification at time 1 and time 2, while (b) those in schools with 30–60% minority students only reported higher national self-identification at time 1.

### Additional Analyses

To check the robustness of the main results, two additional analyses were conducted. First, to better account for measurement error (Kline, 2023), the main analysis was repeated with time 1 and time 2 school belonging specified as latent variables while also correcting for measurement error in the time 1 and time 2 single-item measures of national self-identification. Measurement error in the single-item measures of national self-identification was accounted for by fixing its error variances to (1 - reliability)*variance (Muthén & Muthén, 1998–2017). The error variances were calculated for a Cronbach’s alpha reliability score of 0.86, which represents the average reliability of multiple-item scales of national self-identification used in previous studies with Moroccan- and/or Turkish-origin samples in Belgium, Germany, and the Netherlands (Agirdag et al., 2016; Maes et al., 2014; Martinovic & Verkuyten, 2012). As shown in Table S2 in the online Supplementary Materials, the model correcting for measurement error fully replicated the pattern of within- and over-time associations between national self-identification, perceptions of national fit and school belonging as found in the main analysis. This means that measurement error in the single-item national self-identification measures cannot explain the (non-)findings in the main analysis. With the larger statistical power that comes with correction for measurement error in the single-item national self-identification measures (Phillips & Jiang, 2016), the study findings remain the same.

Second, the vignettes used to assess national fit perceptions of the youth participants specifically described imaginary culturally different peers of *Turkish origin* (see Fig. 1). This begs the question whether perceiving the described peers in the vignettes to fit the national identity holds the same significance for the national identity development of Turkish- as for Moroccan-origin youth. For this reason, and to acknowledge the two distinct ethnic-origin groups in the study sample, a multi-group model was used to test for group differences in the model results across the Moroccan-origin (*N* = 514) and Turkish-origin (*N* = 428) subsamples. The results of the multi-group model are reported in Table S3 in the online Supplementary Materials. In this model, group differences across the Moroccan- and Turkish-origin subgroups were tested for by *imposing equality constraints* on the main associations of interest across the two groups, namely (a) within- and over-time associations of the three perceived national fit measures with national self-identification, and (b) the two cross-lagged paths between school belonging and national self-identification. This constrained multi-group model did not fit the data less well than the unconstrained model, Satorra Bentler ΔChi^2^(8) = 7.875, *p* = 0.446, suggesting that the main results replicate across both origin groups. Additionally, *Wald tests* were conducted to compare the statistical effects of specific associations across the two groups. Non-significant Wald tests further confirmed that the main associations of interest, including the associations of time 1 national fit perceptions with time 1 and time 2 national self-identification, were exactly replicated across origin groups. Thus, the results of this additional analysis suggest that perceiving culturally different peers of Turkish origin in the vignettes to fit the national identity informs the national identity development of Moroccan-origin youth in the same way and to the same degree as that of Turkish-origin youth.

## Discussion

European countries typically demand cultural conformity from immigrant-origin minority persons in exchange for national membership. Against this background, immigrant-origin minority youth across Western Europe face assimilationist pressures and even those who are formally national citizens often struggle to belong socially and to self-identify as nationals of their country of residence (Fleischmann & Phalet, 2018). Given known benefits of national self-identification for psychological well-being (Khan et al., 2020) and civic engagement (Fleischmann et al., 2013), the present study examined the development of minority youth’s national self-identification over one year. Taking the perspective of minority youth, the explanatory focus was on how the school environment as a key developmental context can facilitate the development of their national self-identification. Specifically, this study examined whether and how Moroccan- and Turkish-Belgian youth’s perceptions of cultural difference and national fit in Belgian secondary schools informed their national identity development over and above their evolving school belonging. In doing so, whether school belonging and national self-identification may mutual reinforce each other over time was also explored.

### Perceived National Fit, School Belonging and National Self-Identification

In line with theoretical expectations, varying national fit perceptions significantly predicted the national identity development of the Moroccan- and Turkish-Belgian youth over time, over and above their evolving school belonging. National fit perceptions in this study refer to youth’s perceptions of three imaginary culturally different peers that varied in their degree of cultural difference from the majority group (from *most*, *less* to *least* culturally different). The degree to which they perceived each imagined peer to fit the national identity varied between individual youth, in line with an intersubjective approach of national identity contents as contentious and negotiated in social interactions with others (Chiu et al., 2010). Importantly, those youth who earlier attributed more fit to an imagined most culturally different peer *increased* in their national self-identification one year later. This finding is not an obvious one, as it links the way the minority youth viewed fellow minority peers to how their *own* national identification developed over time. It supports the theoretical argument that perceiving culturally different peers to fit the national identity reflects that the national identity is perceived to be open to cultural diversity. This perception should, and did, enable minority youth to achieve national self-identification via “self-anchoring” (Van Veelen et al., 2016)—specifically, via the anchoring of their own cultural difference in the national identity.

Over and above the earlier perceived national fit of the imagined most culturally different peer, the earlier perceived national fit of the less culturally different peer was unrelated to the Moroccan- and Turkish-origin youth’s national self-identification one year later. Thus, youth participants’ change in national self-identification was driven by the national fit perceptions of the imagined *most* culturally different peer (over and above that of the comparatively *less* culturally different peer); this suggests that attributing national fit in particular to *most* culturally different peers informs the perception of more culturally inclusive national identity contents.

Importantly, the youth participants in this study perceived rather low national fit overall of minority peers, so that not only the imagined most but also the less culturally different peers were not seen as “real nationals”. These descriptive findings illustrate that minority youth are well aware of the prevailing assimilationist societal climate, in which nationals with an immigrant-origin background who are (seen as) culturally different from the majority group, cannot claim to be a “real national”. Still, those youth who perceived slightly more national fit (or less misfit) of the most culturally different peer than others in the study sample, showed a significant increase in national self-identification one year later. This finding implies that even a slight opening of the national identity to cultural diversity can benefit the development of minority youth’s national self-identification.

The fact that national fit perceptions of (most) culturally different peers informed the national identity development of the Moroccan- and Turkish-origin youth *over and above their experiences of school belonging* suggests that minority youth can experience high levels of school belonging through positive intercultural relations with teachers and peers, while still receiving (implicit) messages that they cannot claim to be a fellow national. It supports previous evidence that school-based cultural socialization in Europe tends to refer exclusively to majority cultural knowledge, beliefs, norms, and practices (e.g., Celeste et al., 2019), thereby explicitly or implicitly defining the national identity in ways that exclude cultural difference. Thus, even at high levels of school belonging, the national identity can remain difficult to claim for minority youth due to its restrictive cultural contents.

Still, this study did also explore whether school belonging and national self-identification may mutually reinforce each other over time. It found that the Moroccan- and Turkish-origin youth’s sense of belonging in school was unrelated to their national self-identification over time. In other words, school belonging did not increase national self-identification nor did national self-identification increase school belonging over one year. Given that past research has studied minority youth’s school belonging and national self-identification mostly in separate research streams, this study is likely the first to rigorously test this association over time. Importantly, the non-finding further highlights that overall school communities today do not give minority youth full and equal access to membership in an imagined national community and that ensuring a sense of belonging in school does *not* suffice to facilitate the development of their national self-identification.

Finally, beyond the main findings, the Moroccan- and Turkish-origin youth who earlier viewed the described least culturally different peer as fitting the national identity more, decreased in their school belonging one year later. Note that the *least* culturally different peer was described in the vignette as having adopted customs of the majority culture while not maintaining the customs of her heritage culture. Perceiving such an assimilated peer to fit the national identity more, is likely related to the experience that minority youth’s heritage cultures are not recognized or valued by the majority group; and, previous research has indeed shown that pressure to assimilate negatively affected minority adolescents’ feelings of belonging in school (e.g., Celeste et al., 2019).

### Strengths, Limitations, and Future Directions

To this day, immigrant-origin minority persons often lack a voice in national identity discourse and this social reality is mirrored by a clear gap in scientific research on how minority persons view the national identity (Wiley et al., 2019). This paper addressed this research gap by assessing how minority youth perceive the intersubjective cultural contents of the national identity. Moreover, building on previous correlational evidence (Gharaei et al., 2018), the present study argued and provided first empirical evidence that minority adolescents’ perceptions of the national identity can explain their national self-identification not only within but also over time—over and above evolving school belonging.

Furthermore, a methodological strength of this study is its strong external validity. A stratified random sampling design was used to oversample schools with a high share of immigrant-origin minority students (Phalet et al., 2018); this approach yielded a study sample that included minority participants situated in schools with a very low to a very high share of minority students—thus ensuring optimal external validity. The aim of the present study was to test whether overall perceptions of national fit, school belonging and national self-identification were related over time. As a next step, future studies should examine how these constructs and associations are informed by the school (or classroom) climate as well as their ethnic composition. For instance, first empirical evidence suggests that an actual peer group climate valuing cultural diversity in classrooms with a majority peer presence enables minority youth’s national self-identification (Gharaei et al., 2018) and that schools with inclusive diversity policies promote a greater sense of school belonging in these youth (Celeste et al., 2019). Moreover, school belonging and national self-identification may mutually inform each other only for those minority youth who are in schools that value diversity or who are in schools with a lower share of minority students, where the chance to feel seen and valued or to befriend majority peers is greater (Agirdag et al., 2011).

A limitation of this study is that national self-identification was measured with only a single item. Therefore, measurement error (Muthén & Muthén, 1998–2017) in the single-item measure of national self-identification was corrected for in an additional analysis that fully replicated the main results. In addition, the construct validity of the national self-identification measure was supported by the tested model; most associations, also those with control variables, are meaningful and in line with theoretical expectations. Still, future research could use multiple indicators to capture different dimensions of national self-identification (Wiley et al., 2019) and establish how different dimensions may relate differently to, for instance, perceptions of national fit. Moreover, some of the youth in this study did not respond to the measure of national self-identification (9.8% non-response at time 1 and 7.7% at time 2); thus, future studies could also investigate what may hold minority youth back in answering questions about their self-identification as a national and what this may mean for their national identity development.

Another limitation pertains to the measure of national fit perceptions. Unlike for school belonging and national self-identification, repeated measures of perceptions of national fit over time were not available. Thus, this study did not investigate how stable these perceptions were over time or whether there is a reciprocal relation between minority youth’s perceptions of national fit and their national self-identification. Rather, the study aim was to predict national identity development in minority youth from their earlier national fit perceptions. However, future longitudinal research could use repeated measures of national fit perceptions (a) to examine to what extent (and why) these perceptions of minority youth change over time and (b) to establish causality between national fit perceptions and national self-identification. When going beyond predictive research to establish causality between national fit perceptions and national self-identification, research methods that allow for the disaggregation of between- and within-person effects should be used (Hamaker et al., 2020).

Furthermore, the study findings pertain specifically to Moroccan- and Turkish-Belgian youth. Based on previous research, it seems plausible, however, that the results of this study would generalize to other minority groups as well. A recent European cross-national study has shown, for instance, that factors such as positive contact with the majority group and perceived discrimination explained national self-identification among non-Muslim youth just as well as among Muslim youth (Leszczensky et al., 2020), and the same may hold, for example, for perceptions of national fit: When any (minority) adolescent perceives that someone like them can be a “real national”, it will likely affect the development of their national identity in a positive way. In this vein, the hope is that future research will continue to study the role of national fit perceptions for the national self-identification (and school belonging) of minority youth across various ethnic or religious backgrounds and countries.

In addition, an avenue for future research could be to examine how schools can help reshape socially shared national fit perceptions to be more inclusive of those who are culturally different from the majority group. The results of the present study suggest that overall school belonging alone does not effectively promote the development of national self-identification among minority youth; rather, schools need to address how the national identity is socially defined. However, leaning into conversations about national identity can be difficult for teachers and students alike. Discussions that challenge majority group members’ singular claim on the national identity (e.g., Reijerse et al., 2013) may meet with resistance, and thus potentially exacerbate intergroup tensions in the classroom when minority students’ claims to be real nationals are contested.

Following the example of a Norwegian study by Erdal and Strømsø (2018), future studies could develop and evaluate participatory classroom interventions that simply make students aware of the fact that there is no one clear-cut socially shared definition of the national identity. Erdal and Strømsø (2018) conducted group discussions with the aim to co-construct national belonging among diverse students: They used a piece of string on a table to demarcate the boundary of the national identity and asked the students to place cards with varying identity contents (e.g., “is Christian” or “speaks Norwegian at home”) within, on or outside of the boundary to signify its importance for being truly Norwegian; and, group discussions when collectively placing each card showed that different understandings of the national identity, also among majority students, existed within the group—thereby potentially opening up the national identity to diverse contents in the minds of the students.

## Conclusion

It is well-known that immigrant-origin youth in Western Europe today often struggle to belong socially and to develop national self-identification. Although this negatively impacts their well-being and engagement in society, developmental research on how these youth come to develop national self-identification is still limited. In addition, this limited research work has overlooked how the youth themselves perceive the national identity contents of their residence country. To address this significant gap in research, this study asked how minority youth perceive the national identity and how their perceptions of cultural difference and national fit in their day-to-day school environment relate to their national identity development, over and above their evolving school belonging. While study findings showed that school belonging and national self-identification were unrelated over time, earlier perceived national fit of an imagined (most) culturally different peer uniquely enabled more national self-identification one year later. Thus, this study contributed to existing literature by showing that how minority youth perceive the national identity *matters* for their national identity development. Its findings suggests that schools can promote national identity development in minority youth by redefining the cultural contents of the national identity to include cultural diversity—thereby enabling the youth to perceive that someone like them can in fact fit the national identity. To successfully redefine national identities to include cultural diversity, schools would do well to give voice to the diverse students themselves, and, to develop, implement and evaluate participatory classroom interventions that seek to promote culturally inclusive understandings of the national identity.

## Supplementary Information

Supplementary Materials

## Footnotes

1 In this study, “immigrant-origin minority youth”—or in short: “minority youth”—refers to youth who have at least one parent and/or two grandparents who were born abroad.

2 Belgium has one national school system that is implemented by its three linguistic communities (i.e., in Dutch-, French- and German-speaking schools across the country). Thus, like in other European countries, schools in Belgium can be viewed as national institutions.
