# Supplementary material for: National Identity Development Among Minority Youth: Longitudinal Relations with National Fit Perceptions and School Belonging
Source: J Youth Adolesc. 2024 Jun 19;53(12):2746–61. doi: 10.1007/s10964-024-02036-0 (PMC11534893; doi:10.1007/s10964-024-02036-0)
Supplement: Supplementary file 1 — Supplementary Materials [file 10964_2024_2036_MOESM1_ESM.docx]

National Identity Development Among Minority Youth:

Longitudinal Relations with National Fit Perceptions and School Belonging

**Supplementary Materials**

**Further information on the main measures of this study**

In this study, large-scale survey data from the Leuven-Children of Immigrants Longitudinal Study (Leuven-CILS) conducted in ethnically diverse schools in Flanders, Belgium, was used. The Leuven-CILS project is affiliated with the Children of Immigrants Longitudinal Survey in Europe (CILS4EU; Kalter et al., 2016), and its student questionnaires cover the core modules of the CILS4EU root questionnaire (for the CILS4EU root questionnaire and codebook, see [Documentation CILS4EU](https://deziminstitut-my.sharepoint.com/personal/gharaei_dezim-institut_de/Documents/02_Developing%20national%20self-identification/JYA_REVIEW/Documentation%20CILS4EU)). In the Leuven-CILS student questionnaires minor adjustments of the CILS4EU root questionnaire were made; these include minor changes in item selection and wording (with a view to optimal scale construction), coverage of constructs (prioritizing school over family modules and adding key social psychological constructs) as well as minor changes in ordering and response categories. Detailed information on the Belgian samples, constructs and measures can be found in the Leuven-CILS technical report (Phalet et al., 2018; available upon request). In the following, further information on the main measures of this study, including their translation, is provided.

**National self-identification and school belonging.** The repeated single-indicator measure of national self-identification stems from the CILS4EU root questionnaire and has been validated in previous studies that used the CILS4EU data (e.g., Fleischmann & Phalet, 2018). In contrast, the repeated measure of school belonging represents one of the key social psychological constructs that were newly added in the Leuven-CILS questionnaires. Its four-item scale was adapted from Wang and colleagues (2011) and has since been used successfully in previous research as well (e.g., Celeste et al., 2019).

**Perceived national fit of (imagined) culturally different peers.** The vignette measures of the perceived national fit of (imagined) culturally different peers were specifically designed for the Leuven-CILS project. As shown in Figure 1 of the study, the vignettes describe three imaginary culturally different peers of Turkish origin that varied in their degree of cultural difference from the majority group (from *most*, *less* to *least* culturally different). After reading each vignette, participants were asked “Do you think that … is a real Belgian?”, with responses capturing their national fit perceptions. The vignettes were designed and repeatedly discussed and revised in a culturally diverse research group and network. This process included the invaluable input of academic peers of Turkish descent; and, the names of the persons in the vignettes were chosen in consultation with them - with the aim to use names that are familiar and common amongst this group in Belgium. Additionally, the vignettes (and the full questionnaire) were pretested in qualitative pilot interviews and in sample classrooms with Moroccan- and Turkish-origin students and their feedback was used to finalize them. Note also that the Leuven-CILS vignette measures of national fit perceptions have since been validated in a previous cross-sectional study (Gharaei et al., 2018).

**Translation of measures.** The Leuven-CILS questionnaires were administered in Dutch as the school language and national language in Flanders, Belgium. While vignette measures of perceived national fit were originally designed in Dutch; the national identification question and school belonging items were translated into Dutch from English. All Dutch-language measures were pretested and final item wordings were based on qualitative pilot interviews with minority adolescents of the target age and origin groups.

**Table S1.** Correlations between main study variables (*N* = 942)

|  | 1. | 2. | 3. | 4. | 5. | 6. |
| --- | --- | --- | --- | --- | --- | --- |
| 1. National self-identification T1 | -- |  |  |  |  |  |
| 1. National self-identification T2 | 0.356*** | -- |  |  |  |  |
| 1. School belonging T1 | 0.196*** | 0.108** | -- |  |  |  |
| 1. School belonging T2 | 0.136*** | 0.129*** | 0.437*** | -- |  |  |
| 1. Perceived national fit (*most* culturally different) T1 | 0.170*** | 0.140*** | - 0.045 | 0.028 | -- |  |
| 1. Perceived national fit (*less* culturally different) T1 | 0.138*** | 0.073* | - 0.059^†^ | - 0.007 | 0.522*** | -- |
| 1. Perceived national fit (*least* culturally different) T1 | 0.025 | - 0.017 | - 0.039 | - 0.090** | 0.025 | 0.186*** |

^†^*p* < 0.10, **p* < 0.05, ***p* < 0.01, ****p* < 0.001.

**Table S2.** Results of the longitudinal model explaining school belonging and national self-identification at time 1 and time 2, with (a) school belonging specified as latent variables, and (b) correction for measurement error in single-item measures of national self-identification (*N* = 942)

|  | **Time 1** | | |  | **Time 2** | |
| --- | --- | --- | --- | --- | --- | --- |
|  | School belonging | National self- identification |  | | School  belonging | National self-identification |
|  | *B (S.E.)* | *B (S.E.)* | |  | *B (S.E.)* | *B (S.E.)* |
| **Individual level** |  |  | |  |  |  |
| National self-identification T1 | -- | -- | |  | 0.024 (.050) | 0.316 (.056)*** |
| School belonging T1 | -- | -- | |  | 0.487 (.118)*** | 0.050 (.095) |
| Perceived national fit (*most* culturally different) T1 | - 0.015 (.027) | 0.139 (.042)** | |  | 0.022 (.029) | 0.085 (.042)* |
| Perceived national fit (*less* culturally different) T1 | - 0.014 (.028) | 0.041 (.043) | |  | 0.009 (.030) | - 0.029 (.041) |
| Perceived national fit (*least* culturally different) T1 | - 0.021 (.021) | 0.006 (.036) | |  | - 0.050 (.025)* | - 0.035 (.031) |
| *Controls* |  |  | |  |  |  |
| Age | - 0.040 (.061) | - 0.052 (.055) | |  | - 0.017 (.028) | - 0.018 (.037) |
| Girls | 0.190 (.062)** | 0.289 (.102)** | |  | 0.016 (.057) | 0.106 (.090) |
| Turkish-origin | 0.109 (.067) | 0.004 (.102) | |  | 0.103 (.079) | - 0.115 (.091) |
| Own attitude toward majority culture adoption T1 | 0.046 (.032) | 0.210 (.041)*** | |  | 0.052 (.026)* | 0.136 (.038)*** |
| Own attitude toward heritage culture maintenance T1 | 0.036 (.026) | - 0.086 (.043)* | |  | 0.024 (.027) | 0.019 (.041) |
| **Class level** |  |  | |  |  |  |
| *Controls* |  |  | |  |  |  |
| > 60 % minority in school T1 (ref. cat.) | -- | -- | |  | -- | -- |
| 30-60% minority in school T1 | 0.014 (.181) | 0.222 (.107)* | |  | - 0.015 (.233) | 0.126 (.106) |
| < 30% minority in school T1 | 0.173 (.101)^†^ | 0.396 (.129)** | |  | - 0.020 (.139) | 0.325 (.134)* |
| **Model Fit Indices:** CFI/ TLI/ RMSEA | 0.936/ 0.898/ 0.045 | | |  |  |  |

*Notes.* Unstandardized regression coefficients with standard errors in parentheses are reported. Measurement error in the time 1 and time 2 single-item measures of national self-identification was corrected for by fixing its error variances to (1-reliability)*variance (Muthén & Muthén, 1998-2017) using a Cronbach’s alpha reliability score of 0.86; this Cronbach’s alpha value represents the average reliability of national self-identification scales used in previous studies with Moroccan- and/or Turkish-origin minority samples in Belgium, Germany and the Netherlands (Agirdag et al., 2016; Maes et al., 2014; Martinovic & Verkuyten, 2012).
^†^*p* < 0.10, **p* < 0.05, ***p* < 0.01, ****p* < 0.001.

**Table S3**. Multi-group model comparing youth of Moroccan (*N* = 514) and Turkish origin (*N* = 428)

|  | **Time 1** | | |  | **Time 2** | |
| --- | --- | --- | --- | --- | --- | --- |
|  | School belonging | National self- identification |  | | School  belonging | National self-identification |
| **Moroccan-origin youth (*N* = 514)** | *B (S.E.)* | *B (S.E.)* | |  | *B (S.E.)* | *B (S.E.)* |
| National self-identification T1 | -- | -- | |  | - 0.007 (.034) | **0.210 (.048)***** |
| School belonging T1 | -- | -- | |  | **0.512 (.047)***** | 0.086 (.072) |
| Perceived national fit (*most* culturally different) T1 | - 0.033 (.042) | 0.145 (.063)* | |  | 0.061 (.040) | 0.118 (.055)* |
| Perceived national fit (*less* culturally different) T1 | - 0.011 (.041) | 0.027 (.062) | |  | - 0.011 (.042) | 0.003 (.055) |
| Perceived national fit (*least* culturally different) T1 | **- 0.091 (.034)**** | 0.035 (.052) | |  | - 0.078 (.034)* | - 0.030 (.043) |
| **Turkish-origin youth (*N* = 428)** |  | | |  |  | |
| National self-identification T1 | -- | -- | |  | 0.074 (.044)^†^ | **0.422 (.056)***** |
| School belonging T1 | -- | -- | |  | **0.338 (.062)***** | - 0.046 (.070) |
| Perceived national fit (*most* culturally different) T1 | 0.013 (.044) | 0.130 (.061)* | |  | 0.006 (.048) | 0.045 (.061) |
| Perceived national fit (*less* culturally different) T1 | - 0.054 (.041) | 0.058 (.050) | |  | - 0.007 (.044) | - 0.066 (.052) |
| Perceived national fit (*least* culturally different) T1 | **0.049 (.035)** | - 0.025 (.051) | |  | - 0.038 (.036) | - 0.024 (.047) |

*Notes.* Unstandardized regression coefficients with standard errors in parentheses are reported. In this model, we controlled for students nested in school classes. For simplicity, control variables are not shown; included as controls in the model were age, gender and own acculturation attitudes. Wald tests revealed that only the effect of perceived national fit of the imagined least culturally different peer on time 1 school belonging significantly differed across the two groups, Wald χ^2^(1) = 7.974, *p* = 0.005. Regarding the stability paths, we found that over one year the Moroccan-origin youth were *more* stable in their school belonging, Wald χ^2^(1) = 4.751, *p* = 0.029, but *less* stable in their national self-identification, Wald χ^2^(1) = 8.451, *p* = 0.004, than the Turkish-origin youth in our sample. Differential effects across the two groups are shown in bold.
^†^*p* < 0.10, **p* < 0.05, ***p* < 0.01, ****p* < 0.001.

**References**

Agirdag, O., Phalet, K., & Van Houtte, M. (2016). European identity as a unifying category: National vs. European identification among native and immigrant pupils. *European Union Politics, 17*(2), 285–302. <https://doi.org/10.1177/1465116515612216>

Celeste, L., Baysu, G., Phalet, K., Meeussen, L., & Kende, J. (2019). Can school diversity policies reduce belonging and achievement gaps between minority and majority youth? Multiculturalism, colorblindness and assimilationism assessed. *Personality and Social Psychology Bulletin*, *45*(11), 1603–1618. <https://doi.org/10.1177/0146167219838577>

Fleischmann, F., & Phalet, K. (2018). Religion and national identification in Europe: Comparing Muslim youth in Belgium, England, Germany, the Netherlands, and Sweden. *Journal of Cross-Cultural Psychology, 49*(1), 44–61. <https://doi.org/10.1177/0022022117741988>

Gharaei, N., Phalet, K., & Fleischmann, F. (2018). Contingent national belonging: The perceived fit and acceptance of culturally different peers predicts minority adolescents' own belonging. *Frontiers in Psychology, 9,* Article 1975. <https://doi.org/10.3389/fpsyg.2018.01975>

Kalter, F., Heath, A. F., Hewstone, M., Jonsson, J. O., Kalmijn, M., Kogan, I., & van Tubergen, F. (2016). Children of Immigrants Longitudinal Survey in Four European Countries (CILS4EU) – Full version. Data file for on‐site use. GESIS Data Archive, Cologne, ZA5353 Data file Version 1.2.0, doi:10.4232/cils4eu.5353.1.2.0.

Maes, M., Gonneke, S. W. J. M., & Verkuyten, M. (2014). Perceived ethnic discrimination and problem behaviors in Muslim immigrant early adolescents: Moderating effects of ethnic, religious, and national group identification. *Journal of Early Adolescence, 34*(7), 940–966. <https://doi.org/10.1177/0272431613514629>

Martinovic, B., & Verkuyten, M. (2012). Host national and religious identification among Turkish Muslims in Western Europe: The role of ingroup norms, perceived discrimination and value incompatibility. *European Journal of Social Psychology, 42*(7), 893–903. <https://doi.org/10.1002/ejsp.1900>

Muthén, L. K., & Muthén, B. O. (1998-2017). *Mplus User’s Guide. Eighth Edition.* Muthén and Muthén.

Phalet, K., Meuleman, B., Hillekens, J., & Sekaran, S. (2018*). Leuven-CILS Technical Report Longitudinal* *2012 - 2015.*

Wang, M.-T., Willett, J. B., & Eccles, J. S. (2011). The assessment of school engagement: Examining dimensionality and measurement invariance by gender and race/ethnicity. *Journal of School Psychology*, *49*(4), 465–480. <https://doi.org/10.1016/j.jsp.2011.04.001>
